# Vaccines Induce Homeostatic Immunity, Generating Several Secondary Benefits

**DOI:** 10.3390/vaccines12040396

**Published:** 2024-04-09

**Authors:** Arun B. Arunachalam

**Affiliations:** Analytical Sciences, R&D Sanofi Vaccines, 1 Discovery Dr., Swiftwater, PA 18370, USA; arun.arunachalam@sanofi.com

**Keywords:** cytokine storm, vaccine, innate immunity, adaptive immunity, trained immunity, homeostasis, immuno-wave model, infections, para-specific effect

## Abstract

The optimal immune response eliminates invading pathogens, restoring immune equilibrium without inflicting undue harm to the host. However, when a cascade of immunological reactions is triggered, the immune response can sometimes go into overdrive, potentially leading to harmful long-term effects or even death. The immune system is triggered mostly by infections, allergens, or medical interventions such as vaccination. This review examines how these immune triggers differ and why certain infections may dysregulate immune homeostasis, leading to inflammatory or allergic pathology and exacerbation of pre-existing conditions. However, many vaccines generate an optimal immune response and protect against the consequences of pathogen-induced immunological aggressiveness, and from a small number of unrelated pathogens and autoimmune diseases. Here, we propose an “immuno-wave” model describing a vaccine-induced “Goldilocks immunity”, which leaves fine imprints of both pro-inflammatory and anti-inflammatory milieus, derived from both the innate and the adaptive arms of the immune system, in the body. The resulting balanced, ‘quiet alert’ state of the immune system may provide a jump-start in the defense against pathogens and any associated pathological inflammatory or allergic responses, allowing vaccines to go above and beyond their call of duty. In closing, we recommend formally investigating and reaping many of the secondary benefits of vaccines with appropriate clinical studies.

## 1. Introduction

The immune system is educated to respond to foreign invaders, protecting the host from potential harm, with foreign invaders ranging from inert allergens to harmful pathogens. The immune system should mount an appropriate response to the pathogen so immune homeostasis is maintained. However, despite there being a number of homeostatic mechanisms in place, the cascade of defense reactions unleashed by the host immune system can sometimes become dysregulated, causing harm to the host through a range of complications. Hyperactivation of specific types of leukocytes results in an excessive release of numerous cytokines, which can lead to potentially catastrophic systemic inflammatory or allergic disorders.

The immune system generates innate and adaptive responses when it encounters foreign invaders. The mechanisms that exert innate immune responses can be categorized as constitutive or inducible [1]. A constitutive response is continuous and immediately available, while an inducible response is stimulated, providing an intermediate line of defense between a constitutive and adaptive response. This distinction makes it easy to discern between the rudimentary and naive defensive forces and the sensitive immunological cascades that are potentially dangerous if overstimulated [1]. The inducible innate and adaptive immune responses are critical for both pathogen clearance and maintaining homeostasis. The pro- and anti-inflammatory arms of the immune system are interdependent, keeping each other in check to preserve physiological equilibrium. However, if the equilibrium tilts towards a pro-inflammatory or pro-allergic response, a progressive increase in intensity may lead to a “cytokine storm”, a frenzied release and accumulation of circulating cytokines. This can result in “immune churning”, a tangled and agitated immune response, causing systemic side effects and potential multiorgan failure. One challenge is to determine at which point the expected “normal” inflammatory and associated cytokine response tips over into an abnormal, pathological response. The term “cytokine storm” is used loosely to define acute inflammation or allergy with severe clinical manifestations [2]. It is now essential to establish fingerprints of the different stages of a cytokine storm for prognostic and effective therapeutic approaches. These fingerprints, either qualitative or quantitative, will help to identify the progression of immune responses and thus intervene before they manifest into potential pathological clinical symptoms and death.

This review initially focuses on immune homeostasis and how this is dysregulated by certain infections or allergens, causing inflammatory or allergic pathology and exacerbation of several pre-existing conditions. We then explore the overall effectiveness of vaccines against a variety of infectious diseases, their potential roles in preventing primary and secondary complications of infections, such as associated inflammatory or allergic disorders, and the potential mechanism for these off-label benefits of vaccination. Lastly, we draw conclusions and advocate further research into these additional uses of vaccines that go above and beyond their call of duty.

## 2. Immune Homeostasis

The innate and adaptive immune responses are mediated by various cell types, most of which act as ‘Trojan horses’ that swiftly release numerous soluble factors with varying functionalities and specificities in response to a foreign invader. Therefore, the overall immune response is a non-linear cascade of events with several molecules acting on multiple targets that may be proximal or remote to the molecules. As the immune system has many soluble factors with many overlapping functions, the resulting outcomes are limitless; no single component can be defined as the immunological fulcrum (i.e., the central insurmountable molecule that can tip the immune response towards one outcome or another).

Immune responses are often classified into a small number of archetypal categories—pro-inflammatory, pro-allergic, anti-inflammatory, or pro-humoral—depending on set patterns of effects or consequences. However, it is difficult to clearly define inflammation owing to the complex processes involved [3,4]. Therefore, the category assigned to an immune response should not be considered the exclusive outcome of the response. The classification of innate and adaptive immune responses as pro-inflammatory and anti-inflammatory is based on the types of cells and mediators involved and the effect on the host. Only after the identification of two major classes of T helper cells, type 1 (Th1) and type 2 (Th2), and their associated functions, did this classification gain traction [5,6]. Broadly, Th1 is recognized as the regulator of delayed-type hypersensitivity (pro-inflammatory) reactions and protects the host from intracellular pathogens, and Th2 as the regulator of allergic responses (pro-allergic, anti-inflammatory, or pro-humoral) and protects the host from extracellular pathogens. While CD4+ T-cell subsets may be presented as either pro-inflammatory or anti-inflammatory, these are not mutually exclusive cellular phenotypes due to their plasticity and the discovery of several additional subsets [7].

Generally, when the immune system is stimulated, both pro-inflammatory and anti-inflammatory responses are triggered in parallel. Each response has one or more opposing regulatory components that are mediated by cellular or secreted factors in both the innate and adaptive arms to keep the system in equilibrium. Negative feedback loops operate at specific strengths and times to keep the protective responses effective without completely paralyzing them [8]. In some cases, the functions of effector secretory molecules are tightly self-regulated by the production of inactive precursor forms that are activated upon proteolytic cleavage at a different location in the body [9].

Innate immunity is the first line of defense, performing a preemptive attack on intruders. It relies on a variety of combatants, including macrophages, monocytes, neutrophils, eosinophils, natural killer (NK) cells, innate lymphoid cells (ILCs) and their secretory factors, in addition to physical barriers, antimicrobial peptides, and the complement system. The cells that exert innate immune responses recognize conserved patterns of features on pathogens, referred to as pathogen-associated molecules, ranging from CpG motifs on DNA to formylated methionine on the peptide to surface molecules such as lipopolysaccharide (LPS). Phagocytic macrophages recognize pathogen-associated molecular patterns (PAMPs) on the microorganisms and release a variety of molecules such as type 1 interferons (IFN-α and INF-β), interleukin (IL)-1, and tumor necrosis factor alpha (TNFα), which in turn activate a cascade of pro-inflammatory and adaptive immune responses. Chemokines, released by macrophages, recruit cells such as neutrophils, basophils, eosinophils, mast cells, and dendritic cells to the site of inflammation. Innate immunity is activated quickly and can mount a non-specific recall response with enhanced amplitude and speed, referred to as “trained immunity” [10,11]. Trained immunity or innate memory response can be pro-inflammatory (type 1) or anti-inflammatory (type 2) [12]. The memory response is non-specific in this case and can be triggered by homologous or heterologous stimuli of monocytes, macrophages, type 2 innate lymphoid cells, and NK cells. Transcriptional and epigenetic reprogramming are the molecular basis of innate immune memory and may result in either beneficial or detrimental effects on the host, depending on the context [10]. Type 1 trained immunity can exacerbate pre-existing chronic inflammatory diseases and tissue damage in the host, while type 2 can dampen them. Conversely, type 2 trained immunity can aggravate certain autoimmune or allergy disorders, while type 1 immunity can suppress them [10,12].

Macrophages and dendritic cells are antigen-processing cells. They present antigens and peptide fragments derived from the pathogen to B cells and T cells, leading to the stimulation of an adaptive immune response [13,14] and secretion of antibodies that are specific to each antigen, with a robust pathogen-specific recall response. Antigens can also bind directly to and activate B cell receptors, which are membrane-bound immunoglobulins (Ig) expressed on the surface of B cells. The bound antigen is internalized and processed, and peptides are presented on major histocompatibility complex class I and class II molecules to CD8+ and CD4+ T cells, respectively.

**Figure 1:**
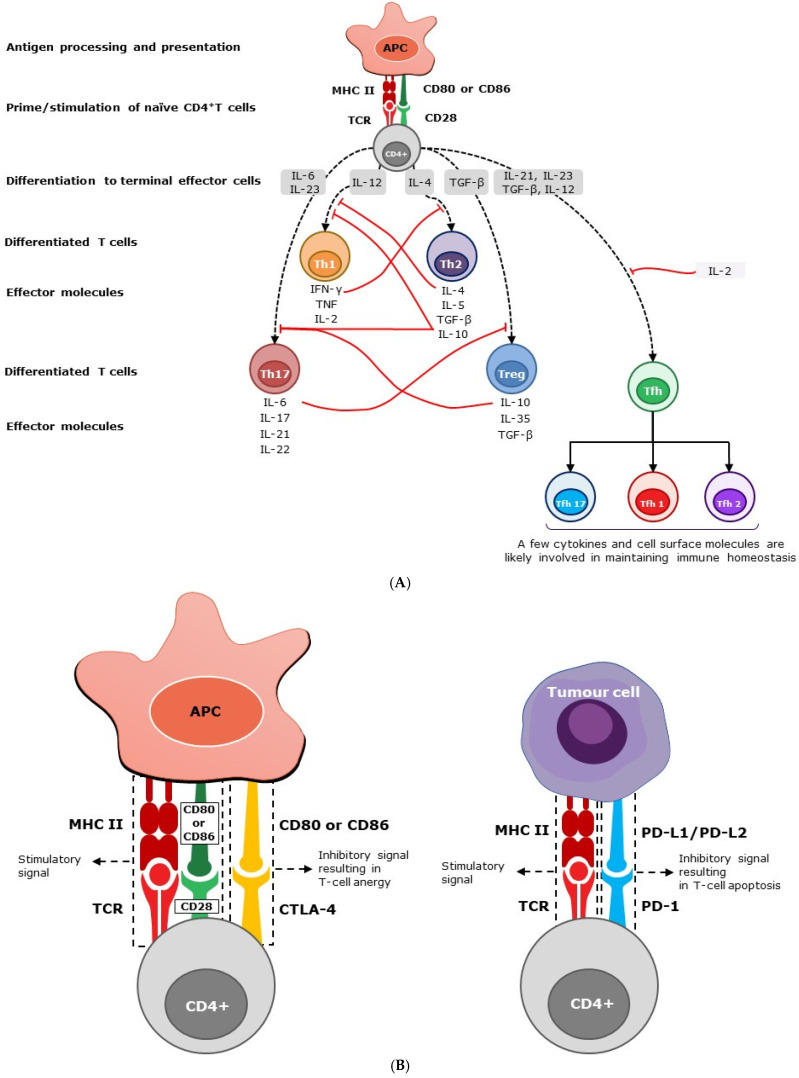
(**A**) Homeostasis of immunity by soluble mediators. Following stimulation, naïve CD4+ T cells mature into different subsets, secreting a variety of soluble cytokines. Effector cells, such as Th1 and Th17, secrete pro-inflammatory cytokines and interleukins, which in turn stimulate a cascade of inflammatory responses. However, effector cells, such as Th2 and Treg, generate anti-inflammatory cytokines, suppressing inflammatory responses. Peripheral Th1 effector cells produce IFN-γ, IL-2, and TNF-α, and predominantly intestine-bound Th17 effector cells secrete primarily IL-6, IL-17, IL-21, and IL-22 cytokines. Th2 effector cells produce IL-4, IL-5, and IL-10, while Treg cells, also known as suppressor T cells, produce IL-10, IL-35, and transforming growth factor–β (TGF-β) predominantly. The level of immune response is determined by the negative (solid red line) or positive (dashed black line) feedback exerted by these mediators. Secreted mediators contribute to immune homeostasis by helping to prevent polarization of the immune system, maintaining an overall balanced response. The abbreviations used are as follows: APC, antigen-presenting cell; CD, cluster of differentiation; IL, interleukin; IFN-γ, interferon gamma; TGF-β, transforming growth factor beta; Th, T helper cell; Tfh, follicular helper T cells; and Treg, regulatory T cells. (**B**) homeostasis of immunity by cell surface molecules. Through TCR, CD4+ T cells recognize peptide fragments of antigens displayed by MHC class II molecules on the surface of cells. In addition to this antigen-specific signal, CD4+ T cells require a co-stimulatory signal through the interaction of CD28 with CD80/86 molecules expressed on APC for their activation. CTLA-4 is a T-cell inhibitory molecule that serves to prevent immunological overreaction [30,31]. While the interaction of CD28 with CD80/CD86 (B7-1/7-2) on antigen-presenting cells promotes T-cell stimulation, the interaction of CTLA-4 with CD80/CD86 suppresses T cells and results in T-cell anergy or exhaustion. CTLA-4 is expressed at the basal level in conventional T cells and constitutively in Treg cells [32,33]. PD-1 is another ‘off-switch’ expressed in T cells. When PD-1 binds to ligands such as PD-L1 and PD-L2, which are expressed on antigen-presenting cells, lymphoid, non-lymphoid cells, and tumor cells, it elicits a signal promoting the apoptosis of antigen-specific T cells [34]. The abbreviations used are as follows: APC, antigen-presenting cell; CD, cluster of differentiation; CTLA, cytotoxic T-lymphocyte-associated protein; MHC, major histocompatibility complex; PD, programmed cell death protein; PD-L1 and PD-L2, PD Ligands 1 and 2; and TCR, T-cell receptor.

Following stimulation, naïve CD4+ T cells mature into different subsets, namely Th1, Th2, Th17, regulatory T cells (Treg) and follicular helper T cells (Tfh), each secreting a different set of cytokines (Figure 1A). However, these subsets are not terminally differentiated and can acquire additional cytokine-producing potential when re-stimulated [7]. Here, we focus primarily on the major cytokines produced and their established functions in humans.

Effector cells such as Th1 and Th17 secrete pro-inflammatory soluble cytokines or interleukins, which in turn generate a cascade of inflammatory responses and events (Figure 1A). However, effector cells such as Th2 and Treg generate pro-humoral (anti-inflammatory) cytokines, which in turn trigger a cascade of humoral responses that suppress inflammatory responses. Th17 cells have been shown to play both pathogenic pro-inflammatory and barrier-protective roles in the intestinal tract [15]. Tfh cells are located predominantly in the germinal center of secondary lymphoid organs, allowing for cognate interaction with B cells and the garnering of B-cell growth and maturation factors, such as IL-4 [16,17]. Extrafollicular or circulatory Tfh cells (cTfh) are phenotypically and functionally comparable to Tfh cells in the germinal center and are monitored as a surrogate to study Tfh cells at this site [18,19]. Based on the preferential expression of chemokine receptors, such as CXCR3 and CCR6, cTfh cells are further subdivided into three major subsets, Tfh1, Tfh2, and Tfh17, which produce some of the characteristic cytokines of Th1, Th2, and Th17 cells, respectively [18,19]. Tfh2 and Tfh17 cells support B cells in producing immunoglobulins (Ig), regulating Ig isotyping, and enhancing Ig affinity [18]. Elevated levels of Tfh cells have been reported in many autoimmune diseases, including multiple sclerosis, systemic lupus erythematosus, rheumatoid arthritis, and myasthenia gravis [20].

Many of the secreted mediators described above, as well as some cell-bound co-stimulatory molecules, are involved in immune homeostasis and pathogen clearance (Figure 1A). Being pluripotent and multi-origin, the cellular phenotypes (pro-inflammatory and anti-inflammatory) of these immune mediators are not mutually exclusive and function simultaneously. The amplitudes of the different types of immune responses are determined by the level of negative or positive feedback exerted by these mediators. Various hypotheses, based on the type, source, and strength of stimulation, have been proposed to explain the factors determining the overall phenotype of an immune response (e.g., pathogen-associated molecular patterns [PAMPs] hypothesis [21], danger model [22], local cytokine milieu [23,24] antigen dose and route of administration [threshold hypothesis [25]]). Several published works support or refute each of these hypotheses, and it is beyond the scope of this review to discuss them in detail.

In general, polarizing the immune response into either pro-inflammatory or pro-allergic phenotypes involves two main stages: early T-cell subset commitment and subsequent subset establishment. Naïve T cells, depending on the nature and source of the stimuli, begin to differentiate into either phenotype and are then fully differentiated and locked in that subset upon constant exposure to certain cytokine milieus (Figure 1A). Maintaining a balanced overall response, both at the subset commitment and the establishment stages, is crucial for immune equilibrium.

IFN-γ, the principal pro-inflammatory cytokine produced by Th1 cells, inhibits the production of the pro-humoral cytokine IL-4 by Th2 cells. Conversely, IL-4 and IL-10 secreted by Th2 cells inhibit the secretion of IFN-γ from Th1 cells (Figure 1A). As a result, they mutually prevent naïve Th0 cells from maturing into Th2 or Th1, respectively [26]. Pro-inflammatory cytokines produced by Th17 cells play a major role in autoimmune diseases such as psoriasis, inflammatory bowel disease, rheumatoid arthritis, and multiple sclerosis (MS) [27]. IL-10 and TGFβ-secreting CD4+ Treg cells suppress heightened pro-inflammatory responses driven by Th17 cells in the intestinal tract and maintain the immune unresponsiveness to self-antigens. As such, Treg and Th17 cell responses counteract each other, maintaining homeostasis and, therefore, suppressing autoimmune responses [27]. Cytokines from Tfh subsets, similar to the cytokines from Th1, Th2, Th17, and Treg cells, are expected to maintain immune homeostasis through a similar feedback mechanism.

In addition to cytokines, cell surface molecules support immunological homeostasis by transmitting inhibitory signals (Figure 1B). These immunological checkpoints or negative regulators, specifically CTLA-4 and PD-1, are essential for balancing immune responses and preventing autoimmunity [28]. These molecules also play an important role in controlling inflammatory tissue damage, such as liver cirrhosis caused by various chronic and acute infections [29]. In conclusion, the soluble factors produced by leukocytes are not target-specific and, as such, they regulate each other through a negative feedback system, promoting immunological homeostasis.

The immune response as a whole can be viewed as a spectrum of response types, where the center of the spectrum is a blend of type-1 and type-2 responses, reflective of immunological homeostasis or the “Goldilocks immunity”, with the extreme ends representing an overall polarization of the immune response to a type-1 or type-2 response. Immunological homeostasis is not, however, an even, static distribution, either qualitatively or quantitatively, of type 1 and type 2 cells and soluble factors. Rather, it is a dynamic equilibrium of responses in constant flux to reach a steady state. Accordingly, Goldilocks immunity consists of a matching combination of type 1 and type 2 responses rather than being dominated by either one. Essentially, the concept proposed here is similar to the Goldilocks conditions (i.e., ‘just right’ conditions) described for T cell development in the thymus [35]. However, an aberrant immune stimulation could bias the cascade of response towards predominantly type 1 (pro-inflammatory) or type 2 (pro-allergic) arms, disrupting immune homeostasis. In this review, we discuss how immune triggers differ from each other and their consequences.

## 3. Disruption of Immune Homeostasis: Cytokine Storms

**Figure 2:**
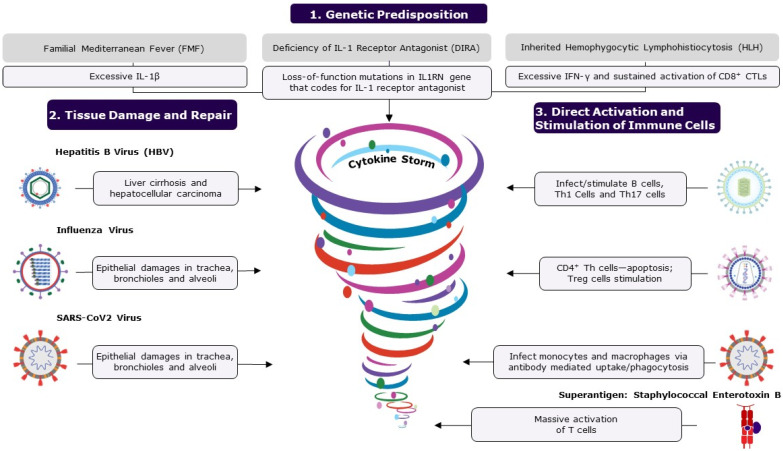
The figure shows the causes of cytokine storms. A cytokine storm is an umbrella term for life-threatening, systemic, inflammatory disorders resulting from elevated cytokine levels, occurring when immune homeostasis is disrupted, causing immune polarization. Polarization to either a type 1 or type 2 response can be caused by several factors, including genetic predisposition, tissue damage and repair, and direct activation and stimulation of immune cells. Microorganisms are the major cause of inflammatory disorders and immune dysregulation. Some infections cause extensive tissue damage and cell death by either direct lysis of cells or apoptosis, triggering the repair process, which becomes dysregulated, disrupting balance. Pathogens such as EBV, HIV, and SARS-CoV-2 can directly attack immune cells, causing over-activation and resulting in increased levels of cytokines. Mostly, the host’s immune system can regulate and prevent the accumulation of pro-inflammatory cytokines; however, an ineffective, over-active response can result in an uncontrolled cytokine storm. If left untreated, excessive levels of circulating cytokines can result in prolonged activation of signaling pathways, causing acute systemic effects such as organ failure and death. The following abbreviations are used: CD, cluster of differentiation; CTL, cytotoxic T cell; IL, interleukin; IFN-γ, interferon gamma; Th, T helper cell; Treg, regulatory T cells; and SARS-CoV-2, severe acute respiratory syndrome coronavirus 2.

Severe disruption to the equilibrium of the immune cascade can lead to a state of relative immune ‘chaos’, resulting in a highly biased, pathological response. Excessive levels of pro-inflammatory cytokines, or a lack of immune balancing regulators in the system, have been shown to underlie a few inherited or acquired inflammatory syndromes [36]. Life-threatening systemic inflammatory or allergic disorders can arise from excessive levels of pro-inflammatory cytokines or pro-allergic cytokines, respectively, collectively referred to as “cytokine storm” or “hypercytokinemia”. However, there is no “calm after the storm”, as a cytokine storm triggers a cascade of events with long-lasting consequences. A number of published reviews have explored this topic extensively [2,37,38]; therefore, this review will provide a brief synopsis of cytokine storms and the associated pathogenic mechanisms relevant to the focus of this paper (Figure 2).

Excessive and exclusive inductions of either type-1 or type-2 responses are equally harmful to the host. While extreme type-1 responses can cause systemic multiorgan failure, excessive levels of type-2 response can lead to immunoparalysis, which is usually not clinically evident but can be detected by decreased leukocyte counts and can promote allergy and autoimmune disorders. Additionally, excess levels of circulatory Tfh cells that primarily secrete type-2 mediators, such as IL-4, are involved in B-cell development, maturation, and antibody production and have been linked to poor prognosis of pre-existing autoimmunity [20].

There are no criteria to identify a cytokine storm before the clinical symptoms appear [2]. Furthermore, the definition of a cytokine storm can be challenging due to the presence of several cytokines with overlapping activities, different potencies and half-lives, their synergistic effect, and their preferential localization in certain organs. In almost all cases, multiorgan failure leads to blood profiling and identification of elevated levels of various pro-inflammatory cytokines in the circulation [2,37,38]. Each type of inflammatory disorder is likely to have one or more cytokines acting as initial mediators, causing a cascade of other pro-inflammatory cytokines to be released [2,37,38]. However, at an early stage of the illness, the inflammatory activities are likely to be orchestrated by a set of key cytokines, which can be identified and quantified to determine fingerprints of the cytokine storm. These fingerprints can help detect and intervene in a potentially harmful immune response before it causes irreversible damage to the host.

### 3.1. Causes of Cytokine Storms

Depending on its nature, intensity and longevity, stimulation, as either a primary or secondary effect, can polarize the immune response to either a type 1 or type 2 response, disrupting immune homeostasis. In some situations, a host can be predisposed to an exacerbated response to a trigger that is otherwise innocuous or tolerable. Here, we discuss such predispositions and the different triggers and their consequences.

#### 3.1.1. Genetic Predisposition

First, we address inherited autoinflammatory diseases to highlight the central role of specific mediators (one or more inflammatory cytokines) in the development of these auto-inflammatory illnesses (Figure 2). Familial Mediterranean Fever (FMF) is a sterile inflammatory disorder characterized by recurrent fever and serositis, with symptoms that include abdominal pain, chest pain, joint pain, and swelling. FMF is caused by autosomal recessive mutations in the *MEFV* gene that codes for pyrin, likely resulting in an excessive expression of IL-1β [39]. Another condition, DIRA (deficiency of IL-1 receptor antagonist), is caused by deleterious mutations in the IL-1 receptor antagonist gene *IL1RN* [39]. Similarly, hemophagocytic lymphohistiocytosis (HLH) is a rare, life-threatening disease caused primarily by the aberrant and sustained activation of CD8+ cytotoxic T lymphocytes [36,40]. The primary form of HLH is inherited, relating to defects in cytolysis, and the secondary form is acquired and triggered by a viral infection like Epstein–Barr Virus (EBV) infection, malignancy such as T or NK lymphoma, or immunostimulants [36,40]. Initial excessive induction of IFN-γ in HLH leads to the secretion of other pro-inflammatory cytokines such as IL-1β, IL-6, and IL-18 [36,40,41]. Therapeutic interventions for these conditions attempt to neutralize or inhibit the synthesis of the key mediators.

#### 3.1.2. Infections Resulting in Tissue Damage

Microorganisms such as viruses and bacteria are the major causes of inflammatory disorders. These organisms inflict, either directly or indirectly, tissue damage in the host (Figure 2). Toxins released by certain bacterial pathogens and the intracellular replication of pathogens can lead to extensive cellular destruction and tissue damage [42]. Cellular death can be caused by either direct lysis of cells or programmed cell death (apoptosis). Most viruses infect cells of a specific organ or system due to tissue tropism, owing to the expression of specific receptors, impairing cells and their functions. For example, the hepatitis B virus (HBV) infects and replicates in hepatic cells, resulting in cirrhosis and hepatocellular carcinoma [43,44]. Similarly, the influenza virus primarily infects and replicates in epithelial cells in the trachea, bronchioles, and alveoli through binding to sialic acid receptors [45]. The effective control of these infections and subsequent recovery from illness depend on the complex interplay between viral and host defense attributes [46]. When the host defense mechanism is in control, the virus and the damaged tissues are cleared from the system in a few days, allowing the body to return to a healthy state. In challenge studies, the viral load of influenza that peaked 2–3 days after inoculation was usually eliminated (undetectable levels) by 6–7 days, and the host recovered to asymptomatic status by 8–9 days [47]. During the wound healing process, myeloid cells, namely macrophages and monocytes, recognize the damage-associated molecular patterns on cells that have been damaged or killed by pathogens and launch a series of events for tissue repair (e.g., recruitment of innate lymphoid cells (ILCs), NK cells, B cells, and T cells) [48]. However, if the pathogen persists in the body, leading to chronic illness, then the wound healing process is likely to become dysregulated with prolonged activation and accumulation of high levels of inflammatory mediators in the circulation, resulting in multisystem disorders [46,49]. At this chronic stage of infection, innate and adaptive immune cells contribute to accumulating pro-inflammatory cytokines. For example, influenza viral infection in some individuals has caused extrapulmonary complications, such as viral myocarditis and viral encephalopathy [50,51,52], and exacerbation of neurodegenerative diseases [53]. Similarly, in severely ill COVID-19 patients, an increased inflammatory response and related cytokine expression are emerging as possible etiologies for respiratory failure [54]. Additionally, severe acute respiratory syndrome coronavirus 2 (SARS-CoV-2) infection has been associated with a significantly higher risk of various autoimmune diseases in a retrospective cohort study, with some racial disparity among the participants [55]. Pathological inflammatory complications associated with influenza, SARS-CoV-2 and other infections have affected a significant number of people in high-risk categories and, despite medical interventions, result in high numbers of fatalities. Similarly, many bacterial exotoxins act either extracellularly or intracellularly, destroying cells or their cellular metabolism [42]. Extensive tissue damage is typically seen in chronic illness and can lead to a cascade of events and flooding of inflammatory cytokines at the repair sites, which in turn often results in fatal systemic inflammatory diseases (Figure 2).

#### 3.1.3. Microorganisms Directly Attacking Immune Cells

Certain pathogens dysregulate the immune response by directly infecting or non-specifically over-activating the immune cells (Figure 2). Some pathogens, such as EBV, human immunodeficiency virus (HIV) and SARS-CoV-2, are known to bind to immune cells, either directly or through antibodies, and stimulate them. EBV is ubiquitous; around 95% of people are infected, usually by early adulthood. EBV has a tropism for B cells and epithelial cells and enters B cells by binding to either complement receptor 1 (CR1 or CD35) or complement receptor 2 (CR2 or CD21). EBV infection has been linked to several autoimmune diseases and cancers [56]. In a longitudinal analysis involving over 10 million young adults, EBV infection increased the risk of developing multiple sclerosis (MS), a chronic inflammatory demyelinating disease, by 32-fold compared to persistent EBV seronegative control group; all these MS cases seroconverted for EBV before the onset of MS, as determined by a specific biomarker, clinical manifestation, or both [57]. Immune dysfunction caused by EBV appears to be the cause of a variety of illnesses. However, the specific mechanism through which EBV infection makes individuals more vulnerable to various diseases is yet to be demonstrated. Several studies suggest that excess levels of circulating Th1 cytokines (IL-2, IL-12, and IFNγ) and Th17 cytokines (IL-17, IL-21, and IL-23) correlate with the pathogenesis of MS, indicating the overactivation of these cell types in MS patients [58]. EBV infection likely results in excessive release of these MS-promoting cytokines. SARS-CoV-2 can infect monocytes and macrophages despite these cells not expressing the viral entry receptor, angiotensin-converting enzyme 2 (ACE2). A recent study demonstrated that antibody-bound SARS-CoV-2 viruses were internalized by monocytes and macrophages that express Fcγ receptors through antibody-dependent phagocytosis [59]. A molecular examination of the mechanism revealed that non-fucosylated antibodies, found during acute infection but not after vaccination, facilitated virus uptake and infection of the cells [59]. Such a viral infection may activate cellular inflammasomes, supramolecular complexes of innate immune sensors in the cytoplasm of activated immune cells that activate inflammatory caspase and adaptor protein [60], resulting in deleterious immunological reactions and multiorgan failure. Similarly, HIV infects CD4+ T helper cells and produces a slew of immune system malfunctions [61]. While the impact of HIV on the immune system is not yet fully understood, the direct interaction of envelope glycoprotein gp120 and CD4 renders primed CD4+ T helper cells susceptible to activation-induced apoptosis while promoting Treg subset survival. Superantigens such as staphylococcal enterotoxin B (SEB) are potent T-cell mitogens that can non-specifically stimulate polyclonal T cells, resulting in massive systemic activation of T cells and the release of pro-inflammatory cytokines [62]. The enterotoxins produced by *Staphylococcus aureus* and *Streptococcus pyogenes* can directly bind T cell receptors (TCRs), bypassing antigen processing and presentation. When stimulated with superantigens, mature CD4+ and CD8+ T lymphocytes proliferate and overwhelm the surrounding environment with pro-inflammatory cytokines, resulting in severe systemic pathologies [62]. In conclusion, a small number of pathogens can directly attack immune cells and cause immunological dysfunction by activating and depleting immune cells, as well as changing their functions, resulting in a variety of diseases.

## 4. Vaccination

Unlike the infections and tissue damage discussed above, most immune responses elicited by vaccinations or controlled infections contain a mix of both pro-inflammatory (i.e., type 1) and anti-inflammatory (i.e., type 2) elements, thereby maintaining the overall immune homeostasis. Here, we focus the discussion on the protection and other benefits offered by different vaccines, as well as the likely molecular mechanisms underlying this.

### 4.1. Protection from Specific Pathogens

**Figure 3:**
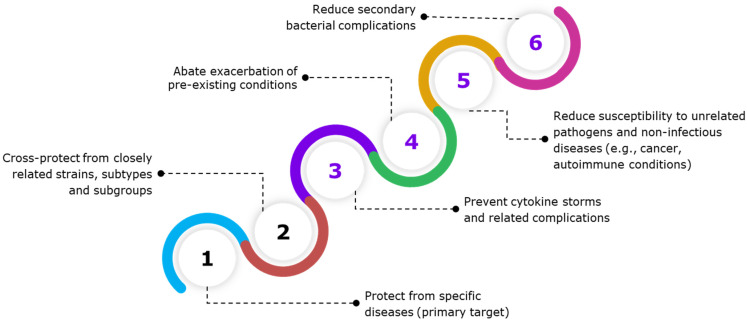
The figure shows the specific and para-specific effects of vaccines. Vaccination often elicits a controlled immune response, containing a mix of pro-inflammatory and anti-inflammatory cytokines, thereby maintaining immune homeostasis. Beyond protecting against the target pathogen, vaccines also exhibit other immune benefits. Some vaccines may confer protection against closely related pathogens that share conserved antigenic epitopes. However, vaccines have also shown para-specific effects, which are non-specific or heterologous effects that protect or reduce host susceptibility to unrelated infectious and non-infectious diseases. Para-specific effect (PSE) of vaccines prevents or lowers the host’s susceptibility to secondary bacterial infections, sepsis, aggravation of pre-existing conditions, and non-infectious diseases, such as cancer and cardiovascular diseases, allergic asthma, as well as autoimmune diseases, such as type 1 diabetes.

Vaccines are designed to elicit a specific immune response that protects hosts from a specific pathogen. This is generally accomplished through a targeted adaptive immune response involving specific T and B cells. Functional antibodies that are specific to the pathogen or its pathogenic element (e.g., toxins) are primarily responsible for conferring protection against the targeted pathogen (Table 1). These immune responses reduce the likelihood of the progression of infections, thereby reducing the likelihood of potential cytokine storm and associated pathological consequences (Figure 3). Thus, the effectiveness of many vaccines can be assessed using the levels of specific functional antibodies as correlates of protective immunity. Depending on the pathogen or pathogenic component, a protective antibody response may be evaluated based on the neutralizing, bactericidal or opsonizing activity of the antibodies generated. However, predisposing factors in the host, namely host genetics as well as environmental and gut microbiome factors [63], can tip the balance towards either a type 1 (pro-inflammatory) or type 2 (pro-allergic) immune response.

### 4.2. Cross-Protection from Closely Related Pathogens

Some antibody responses may also offer protection against closely related pathogens that share a few conserved antigenic epitopes (Figure 3 and Table 1). For example, the cowpox vaccine containing *Variola vaccina* also confers protection against *Variola major* infection, whereas the Bacillus Calmette–Guérin [BCG] vaccine containing *Mycobacterium bovis* confers protection against *Mycobacterium tuberculosis* infections in humans. A number of other viral and bacterial vaccines have been shown to provide variable levels of cross-protection against strains, types, or groups of organisms that are closely related to the target pathogen [64]. Both epidemiological and experimental evidence also suggest that pre-existing cross-reactive cellular immunity evoked by human coronaviruses, such as common cold coronaviruses, may modulate COVID-19 severity and the response to COVID-19 vaccinations [65]. Subsequent COVID-19 severity is therefore reduced, and both the amplitude and functional avidity of the T-cell response to COVID-19 vaccines are increased [66]. However, the role of cross-reactive antibodies in modulating COVID-19 severity and the immune response to COVID-19 vaccination is unclear. Similarly, mice immunized with a cold-adapted influenza virus (X-31 ca, H3N2), especially at a higher dose, were shown to be fully protected against heterosubtypic and heterotypic influenza viruses [67,68,69].

Despite technical and regulatory obstacles, there is renewed interest and effort in developing universal influenza vaccines of broad specificity, especially in the wake of the coronavirus pandemic [70,71,72,73], and efforts to develop vaccines with broad specificity protecting the host against potential future influenza virus strains are ongoing [74,75]. Indeed, unique features of hemagglutinin antigens in a recombinant influenza vaccine may improve this additional benefit of cross-protection against other strains within the relevant influenza subtype or lineage [76]. While many vaccines licensed for the prevention of an intended disease have also been found to cross-protect against closely related pathogens, it should be noted that their use for the latter would be considered as ‘off-label’.

### 4.3. Para-Specific Effects of Vaccines

The protection conferred by vaccination often extends beyond preventing or reducing the primary disease (Figure 3). Pathogen-driven secondary complications, such as systemic inflammatory disorders and associated autoimmune diseases, may also be prevented by certain vaccines. A growing number of independent studies have demonstrated that vaccination may not only provide protection against specific diseases but also induce non-specific or heterologous effects that protect or reduce the susceptibility of the hosts to a small number of unrelated pathogens, autoimmune diseases, and pre-existing conditions [77,78]. To refer to these off-label benefits of vaccinations, we use the term “para-specific effect” (PSE) to avoid the potential negative connotations of the term “non-specific effect” (NSE), as referred to by a few groups [77,78].

Smallpox and BCG vaccines have been shown to protect or reduce susceptibility to other diseases, namely measles, whooping cough, and scarlet fever, lowering the risk of hospitalization and mortality [79,80,81]. These investigators further demonstrated that the observed PSE of vaccines was due to the generation of specific cytokines and the activation of a favorable innate immune response in addition to cross-reactive antibodies and T cells (Table 1). The live attenuated BCG vaccine, while primarily recognized as the tuberculosis vaccine, is also indicated for treating and reducing the recurrence of urinary bladder cancer, an unrelated illness, due to its ability to induce para-specific immunity [82]. Recent studies additionally demonstrated the beneficial effects of the BCG vaccine for treating Type 1 diabetes [83]. Using the Alzheimer’s disease (AD) biomarker Amyloid Probability Score, Dow et al. (2022) recently demonstrated that the BCG vaccination also significantly decreased the probability of developing Alzheimer’s disease in a high-risk group [84].

Immunization with cold-adapted influenza virus (X-31 ca) generates short-term cross-protection against respiratory syncytial virus (RSV) challenge, which was not mediated by antibodies [27]. The observations of increased levels of plasmacytoid dendritic cells, IFNγ, and IL-6 cytokines, together with the absence of cross-protection in Toll-like receptor (TLR) 3/7 knockout mice, indicate that RSV clearance was caused by a moderately activated innate immune response (Table 1) [27]. Although influenza is largely a respiratory illness that may lead to pneumonia, evidence also links it to acute cardiovascular disease (CVD), such as acute myocardial infarction and stroke [85,86,87]. A significant association with CVD is not observed in some other respiratory illnesses, such as RSV or parainfluenza virus infections [87,88]. During influenza seasons in the United States, influenza appears to increase symptoms of CVD, which accounts for 71–85% of mortality in elderly populations aged ≥65 years [89]. The influenza virus causes CVD both directly through viral infection and indirectly through inflammatory cells and mediators [90]. The presence of the influenza virus in the aorta suggests potential infection-induced damage to cardiovascular tissues and subsequent vascular hyperpermeability [91,92]. Following influenza infection, increased recruitment of inflammatory macrophages into the aortic arch may promote atherosclerotic lesions [93]. Infection-induced pro-inflammatory cytokines, namely IFNγ and TNFα, are associated with unstable plaques, which may then be dislodged, resulting in occlusion [94]. These pro-inflammatory cytokines have also been shown to exert arrhythmogenic effects directly on cardiac cells, resulting in cardiac fibrosis and electrophysiological changes, such as the instability of myocardial electrical impulses [95]. A number of studies, including meta-analyses, have shown that influenza vaccines confer protection against specific respiratory infections and the accompanying non-specific CVD [96,97,98,99,100]. It is worth noting that the effectiveness of influenza vaccination in reducing cardiovascular-related mortality is comparable to that of therapeutic medications like statins, β-blockers, and ACE inhibitors [101,102]. Many studies found protective PSEs of influenza vaccination against severe COVID-19. Before the introduction of COVID-19 vaccination, the severity of COVID-19 and related hospitalization was estimated to drop by >85% in influenza-vaccinated healthcare workers compared with unvaccinated healthcare workers [103]. In another study, individuals who received an influenza vaccine two to six months before testing positive for SARS-CoV-2 had a significant reduction in sepsis, stroke, and intensive care admissions compared with unvaccinated individuals 60 days after a positive SARS-CoV-2 diagnosis [104]. Protection against SARS-CoV-2 infection has also been observed following vaccination against other diseases, including polio, measles, mumps, rubella, varicella, hepatitis A, hepatitis B, *Haemophilus influenzae* type b, and *Streptococcus pneumoniae* when received within the previous five years prior [105]. These findings validate the protective PSEs of vaccination against unrelated diseases.

Influenza vaccines also protect against secondary bacterial complications and reduce the severity caused specifically by *Streptococcus pneumoniae*, *Staphylococcus aureus*, *Streptococcus pyogenes*, and *Haemophilus influenzae*, which account for 40–95% of influenza-related mortality in previous flu pandemics [106]. Influenza vaccines prevent the viral-mediated killing of alveolar macrophages and impairment of NK cells, which are believed to be responsible for the off-target effects of influenza vaccines (Table 1) [107].

Similar to influenza, the SARS-CoV-2 virus is known to cause extrapulmonary complications, including CVD and multisystem inflammatory syndrome (MIS) due to systemic hyperinflammation induced by the virus [108]. COVID-19 vaccination significantly reduced the risk of acute myocardial infarction and ischemic stroke after SARS-CoV-2 infection [109].

Live attenuated rotavirus vaccines reduced the incidence of type 1 diabetes in children under 4 years of age [110,111]. Additionally, vaccines derived from mycobacteria (e.g., BCG) and helminths have been proposed to prevent allergic asthma and other allergic diseases [112]. Remarkably, a recent meta-analysis showed that routine adult vaccination is associated with a reduced risk of future dementia by 35%, which was vaccine dose-dependent, and the reduction was significant with influenza, herpes zoster, tetanus-diphtheria-acellular pertussis, hepatitis A, hepatitis B, typhoid, and rabies vaccinations [113]. Live attenuated vaccines such as the measles, mumps, rubella (MMR) vaccine and the oral polio vaccine (OPV) have been observed to improve the resistance to pneumonia and sepsis [114].

A few groups have observed female-biased differences in PSEs [114]. PSE induced by live vaccines, such as measles, BCG and smallpox vaccines, is more pronounced in females than in males, which could be partly due to the overall stronger immune response elicited in females [114]. While several vaccines have been observed to induce protective PSE, a few inactivated vaccines, such as diphtheria tetanus pertussis (DTP), hepatitis B virus (HBV) and DTP combo with HBV, inactivated polio and *H. influenzae* type b vaccines, have been found to generate a detrimental PSE, which are more pronounced in female infants than male infants [115]. The higher mortality of young girls than boys, in general, would have offset PSE in young girls [114].

To conclude, many vaccines have been observed, primarily through observational studies and meta-analysis, to induce PSEs that reduce or prevent potential secondary complications by the target pathogen and render the host less susceptible to a small number of unrelated pathogens, autoimmune diseases, and pre-existing conditions. The potential mechanisms of para-specific vaccination are summarized in Table 1.

### 4.4. Novel Mechanism of Para-Specific Protection by Vaccination

#### 4.4.1. Goldilocks Immunity

Vaccines prepare the immune system ‘armed to the teeth’ with the recall response needed to protect from specific diseases. Vaccine-induced specific immunity prevents or eliminates the pathogen before it takes over the host. Generally, vaccines induce ‘Goldilocks immunity’, i.e., a balance of type-1 (IgG1, IgG3 and major type-1 cytokines and cytotoxic T lymphocytes [CTLs]) and type-2 (IgG2, IgG4, and major type-2 cytokines) immune responses, comprising both innate and adaptive immunity [116,117,118,119]. The combination of cellular plasticity and the activation of different cellular subsets in parallel is likely responsible for the accumulation of both sets of type-1 and type-2 cytokines in the milieu. It has previously been demonstrated that activated naïve CD4+ T cells (Th0) release both type-1 and type-2 cytokines during the first eight cell divisions occurring within 5 days, with type-1 cytokines released at the beginning and type-2 cytokines at the end of the differentiation cycle [120]. Such cellular plasticity is expected before the terminal differentiation of lymphocytes, resulting in the accrual of both sets of cytokines throughout the early phase of the immune response. The antigens and immune-potentiating adjuvants included in vaccine formulations, which can trigger a variety of cellular subsets, are equally responsible for this balanced immune response. Typically, vaccines contain one or more core antigens from the pathogen that induce a protective immune response in the host and may also contain residual levels of other pathogen-derived proteins [119]. Based on their immunogenic vigor, these vaccine components could stimulate a variety of cellular subsets of innate and adaptive immunity arms.

#### 4.4.2. Immuno-Wave Model

**Figure 4:**
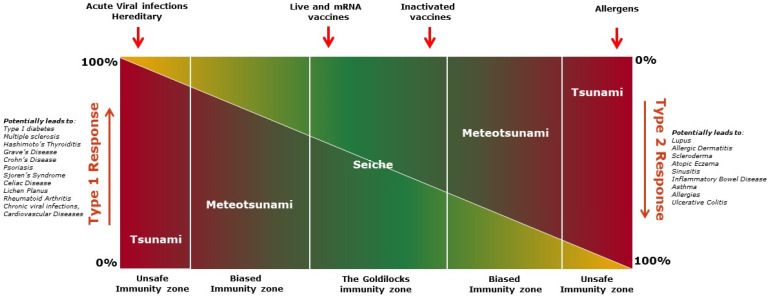
The figure shows immuno-wave model describing spectrum of immune responses. The novel immuno-wave model displays the dynamic nature and various spectra of immune responses ranging from an equilibrium zone to a highly polarized zone of type 1 or type 2 immune responses. Type 1 and Type 2 response levels are represented by the color spectrum, with red being the strongest response and yellow the lowest. In the Goldilocks zone, the immuno-wave is bidirectional and not progressive enough to harm the host. Fluctuation to either end of the Goldilocks zone is minimal, or a shallow ‘seiche wave’, with type 1 and type 2 immunity counteracting one another, and does not result in severe adverse events. However, polarization to either type of immune response enters into unsafe immunity. Polarization can be caused by ‘seismic activity’, such as tissue injury, resulting in a tsunami-like wave. This unsafe immunity zone can cause allergies, autoimmune diseases, multi-organ failures, and death. In the immuno-wave model, vaccines elicit a seiche wave-like immunity, balancing type 1 and type 2 immune responses, resulting in a ‘quiet alert’ state of the immune system in the Goldilocks zone.

An induced immune response may be predominantly type 1, type 2, or a blend of both, progressing from one to another at different stages. Different types of water waves are referred to here as analogies to illustrate distinct features of various stages of the immune response, creating an immuno-wave model (Figure 4).

Goldilocks immunity, containing both type-1 and type-2 immune responses, is a dynamic equilibrium of responses attempting to achieve a steady state. Both type-1 and type-2 responses oscillate back and forth with decreasing amplitude until the response reaches an equilibrium state, like a “Seiche” standing wave [121,122] (Figure 4). In the Goldilocks immunity zone, neither the type-1 nor the type-2 response is exclusively observed, although one response might moderately outweigh the other at either end of the zone (Figure 4). In general, this type of response wave is bidirectional and not progressive enough to harm the host. The fluctuation between type 1 and type 2 immune responses is shallow, and it is seldom noticed clinically as it does not translate to severe adverse events. At this parley zone, the presence of both type-1 and type-2 immunity counteracts one another, acting as the restoring force (like gravity acts on the seiche wave), preventing pre-eminence of one arm over the other, keeping each in balance.

Vaccines and controlled or latent infections, like powerful winds, primarily evoke a seiche-like immune wave in the host, maintaining the overall balance of immune responses and thus preventing the rise of any potential hegemon. Whereas acute infections, with the rapid expansion of pathogens or anaphylaxis, like fast-moving storms, can cause a meteotsunami-like wave [123], forcing the immune response into unsafe or biased immunity zones (Figure 4). This type of immune wave may be exacerbated by ‘seismic activity’ such as extensive tissue injury, resulting in the development of a tsunami-like wave [124], causing inflammatory multi-organ failures, allergies or autoimmune diseases, and permanent damage to the host (Figure 4). These immune waves, like the meteotsunamis or tsunami waves, are unidirectional and may progress to a point of no return if left unchecked in the early stage. This may be avoided and balance may be restored at the meteotsunami stage if damage to the host is minimal. At the tsunami stage, however, damage to the host is substantial and likely to be untreatable or even fatal. In essence, “violence begets violence”; immune vigor corresponds to the intruder’s aggression, and as a result, the host suffers. Vaccines, according to the proposed “immuno-wave” model, handle the immune system diplomatically and induce a specific Goldilocks immunity, balancing type-1 and type-2 immune responses, resulting in a ‘quiet alert’ state of the immune system, which leaves fine imprints of both pro-inflammatory and anti-inflammatory milieus in the body. The resulting balanced milieu keeping a check on the potential surge of each type of immune response is likely responsible for the PSEs of vaccines (Table 1).

#### 4.4.3. Tailored Immune Responses to Vaccines

According to Cohen (2000), the reactive immune system recognizes the antigen, constructs an image of it, known as “correspondence,” and then develops an appropriate response that involves continuous immunological dialogue with the body [125]. In this process, the intensity of the immune response is determined by the type and the level of immune trigger. Vaccines are designed to contain the optimal level and type of antigen to evoke a tailored immune response before the antigen is rapidly cleared from the system [126,127,128,129]. Levels of functional IgG antibody subclasses (IgG1, IgG2, IgG3 and IgG4) produced primarily through the distinctive molecular mechanisms of the type-1 and type-2 arms are considered to serve as direct or surrogate immune correlates of protection [117,119,130,131,132]. Vaccines containing live attenuated organisms or nucleic acid strands coding for the target antigens mostly tend to evoke a balanced immune response or a response that is slightly biased towards type 1 [133,134,135,136], falling to the left of the Goldilocks immunity zone (Figure 4). Inactivated vaccines also tend to induce a balanced immune response but, in some cases, with a slight bias towards a type-2 response, falling to the right of the Goldilocks immunity zone (Figure 4). Almost all parenterally administered vaccines contain inactivated organisms or components of an organism, such as proteins or polysaccharides that are derived from the pathogen or produced synthetically to induce protective immunity. Specific virulence factors, such as bacterial toxins, may be inactivated by chemical treatment or mutagenesis techniques for inclusion. Other structural and non-structural elements of the pathogen, such as lipopolysaccharides in Gram-negative bacteria, superantigens such as enterotoxin in bacterial and viral pathogens, and other moieties that are dangerous signals to the immune system, are also eliminated from the vaccine [137]. In this way, vaccines contain only the relevant antigens at the ideal dosage optimized by clinical studies, which is required to generate the appropriate immune response.

The immune system has a heterogeneous mixture of cells and soluble factors dispersed throughout the tissues and in the blood, resulting in varying gradients of cells and soluble effectors depending on the location. Hence, the evaluation of blood samples or specific tissue samples, while providing molecular insight into various aspects of the immune system, often leads to a restricted view of the immune response that is not reflective of the system as a whole [138]. Results from such studies should be interpreted with caution because they are likely to be biased toward one or a few specific types of immune response. Using a systems immunology approach to study multiple relevant tissues would likely provide a broader and more integrated perspective of the immune response elicited against pathogens and vaccines [138,139]. This approach can also demonstrate the potential role of other systems, such as metabolic pathways, in achieving an optimal immune response [140,141]. Thus, when examined holistically, most immune responses induced by controlled infections or vaccination comprise a mix of both pro-inflammatory (i.e., type 1) and anti-inflammatory (i.e., type 2) factors, as evidenced by the absence of severe adverse reactions and subsequent disorders typically associated with skewed responses.

Using the systems vaccinology approach, Nakaya et al. (2011) showed that the use of inactivated trivalent influenza vaccine (TIV) and live attenuated influenza vaccine (LAIV) over the course of three successive influenza seasons induced innate and adaptive immune responses in the blood and peripheral blood mononuclear cells, with distinct molecular signatures [142]. LAIV, similar to the live attenuated yellow fever virus vaccine (YF-17D), primarily activated several interferon-related genes, whereas the TIV enhanced the expression of genes related to immunoglobulin [142,143], explaining the high frequency of plasmablasts and a sixfold increase in the hemagglutination inhibition (HAI) titers observed with TIV compared with LAIV [142]. Similarly, an inactivated split influenza vaccine predominantly stimulated a Th2 response following both the priming and booster vaccinations in mice [144], whereas the corresponding whole virus influenza vaccine induced a Th1 response upon priming, and a combination of both Th1 and Th2 responses following booster immunization. Detailed phenotypical analysis revealed that there was a significant correlation between the transient emergence of Tfh-like cells (expressing IL-2, IL-10, IL-21, and IFN-γ) 7 days after influenza vaccination and increased pre-existing protective antibody response [118]. Similarly, Pilkinton et al. (2016) observed a strong correlation between transient activation of inducible T-cell costimulator+ cTfh cells, which support B cell maturation, and seroconversion in adults following influenza vaccination [145]. This trend was greater with the high-dose influenza vaccine than with a standard-dose vaccine in older adults. It is important to note that the emergence and activation of cTfh cells following influenza vaccination were transient in nature [118,145,146]. These results indicate that LAIV and inactivated split influenza vaccines were able to evoke Goldilocks immunity, marginally favoring type 1 or type 2 responses, respectively.

Using blood transcription modules developed through integrating large-scale data, Li et al. (2014) demonstrated that different classes of vaccines induced similar blood transcriptome response signatures, along with distinct molecular signatures of type 1 and type 2 antibody responses [131]. In the study by Li et al., both the meningococcal polysaccharide conjugate vaccine and trivalent inactivated influenza vaccine predominantly induced genes related to the B-cell antigen receptor signaling pathway. However, the live attenuated yellow fever vaccine (YF-17D) and LAIV induced genes related to interferon and T-cell antigen receptor signaling. Interestingly, the polysaccharide antigens in the meningococcal vaccine induced the maturation of dendritic cells and the expression of IL-6, TNF, costimulatory molecules and complement [131]. Cytokine analysis of peripheral blood mononuclear cells obtained from YF-17D vaccine recipients and stimulated in vitro with Yellow Fever virus showed comparable levels of IFNγ-secreting Th1 cells and IL-4-secreting Th2 cells [147]. Similarly, a systems vaccinology approach evaluating immune responses to 13 different vaccines revealed that live vaccines consistently elicited early adaptive and delayed innate responses [130]; in contrast, inactivated vaccines elicited early innate responses. These findings highlight the value of evaluating immune response kinetics to define distinct immunological signatures of vaccines [130].

Many live attenuated vaccines, such as LAIV, rotavirus vaccine and OPV, are administered via non-parenteral (intranasal and oral) routes. The live attenuated viruses in these vaccines stay in the body for a longer period than inactivated vaccine antigens delivered via injection and are required to break through several mucosal barriers in order to trigger an adequate antibody response in the mucosal membrane of the respiratory or digestive system, where the pathogen and disease persist. Moreover, the live attenuated vaccine viruses by selection do not cause the extensive tissue damage seen with wild-type viral infections and, thus, generally do not induce cytokine storms or other deleterious immune reactions in the host. The COVID-19 mRNA vaccine BNT162b2 was shown to induce both type 1 (e.g., IFNγ) and type 2 (e.g., IL-4 and IL-10) cytokines after booster vaccination, with type 1 being more dominant [148]. However, the upregulation of antibody-secreting cells observed on day 7 following vaccination with inactivated seasonal and AS03-adjuvanted pandemic influenza vaccines and live viral vector Ebola and HIV vaccines was not observed with BNT162b2.

In latent infections, there is an equilibrium between immune control and pathogen persistence. For example, *Mycobacterium tuberculosis* remains latent in most infected individuals, with bacteria contained in macrophages in granulomas infiltrated with innate and adaptive immune cells expressing similar levels of both type 1 and type 2 cytokines [149,150]. However, the latent *M. tuberculosis* infection, in a subset of BCG-vaccinated infants that maintain a balanced immune response, can progress into an active infection, which, in turn, elevates the levels of IFNγ, inflammation, and immune activation, resulting in a type-1 skewed immune response [150,151]. While the cause-and-effect relationship is unclear in this situation, the progression from latent to active infection highlights the importance of a controlled immune activation, such as that provided by vaccines, for a balanced response.

With many acute infections, intense antigenic stimulation persists, continuously stimulating immune cells. As such, long-lasting, intense stimulations result in high quantities of pro-inflammatory cytokines, disrupting homeostasis. Excess levels of pro-inflammatory cytokines, such as TNF, IL-1β, IL-12, IL-18, and the activation of the complement system in sepsis eventually lead to inflammatory syndrome with serious consequences (Figure 4) [152]. Vaccinations, in contrast to acute infections, deliver a controlled stimulation of immune cells, which results in a balanced type 1 and type 2 response (Figure 4). In conclusion, vaccines generate a Goldilocks immunity, with a slight bias in either direction in some situations. Such a modest bias, however, is not exacerbated by the transitory triggering of the immune system intrinsic to vaccination and is promptly restored by the presence of both type 1 and type 2 counteracting factors.

### 4.5. Adjuvants

Fast clearance of vaccine antigens from the system may not be ideal for the maintenance of long-term immune memory [126,127,128,129]. However, rapid clearance can be mitigated by using adjuvants and revaccinating individuals at regular intervals [129]. The inclusion of adjuvants or nanoparticles in vaccines helps to release the antigens slowly and induce immune responses steadily over a longer period.

Recently, several adjuvants have been licensed as immunopotentiators of vaccines, and they work through antigen delivery, direct stimulation of the innate immune system, or both [153,154]. Most adjuvants were found to enhance antibody response by stimulating different innate receptors, such as TLRs, and recruiting leukocytes at the site of injection. Aluminum salts (alum) are widely used as adjuvants and were thought to enhance antibody production by promoting type 2 immune responses [155,156]. However, recent system vaccinology studies and other conventional studies indicate that the observed type 2 bias is unlikely to be attributable to alum’s direct action [157]. Adjuvant system 04 (AS04) and Alum/TLR7 are alum-based adjuvants with a TLR agonist, and they were found to enhance antibody response by promoting type 1 responses [154]. MF59 is an oil-in-water emulsion adjuvant containing squalene stabilized in non-ionic surfactants, evoking both type 1 and type 2 responses [158]. As most adjuvants recruit a wide range of immune cells that secrete a variety of cytokines at the injection site, it is difficult to categorize adjuvants as solely type 1 or type 2 inducers. Immunopotentiation by adjuvants at the expense of safety is unacceptable; therefore, the development of a new generation of adjuvants should focus on inducing a balanced immune response, as seen with the majority of the adjuvants currently in the market [159].

### 4.6. Non-Immunomodulatory Effects of Cytokines

The mixture of type 1 and type 2 cytokines in the Goldilocks immunity zone, induced by most vaccines, have several immunomodulatory and non-immunomodulatory effects, culminating in the observed PSEs. The inhibitory effect of type 1 cytokines, such as IFN-γ, on Th2-cell differentiation and of type 2 cytokines, such as IL-4 and IL-10, on Th1-cell differentiation, are discussed earlier in this review. A few type 2 cytokines have cellular compensatory mechanisms to dampen inflammation (Table 1). Cytokines such as IL-19 reduce inflammation by inhibiting the expression of adhesion molecules, therefore decreasing the infiltration of leukocytes and promoting angiogenesis [160]. Similarly, the induction of granulocyte colony-stimulating factor (G-CSF) by the BCG vaccine was found to stimulate neutrophil generation, which in turn provides non-specific protection against sepsis [161]. IL-10-producing CD5+ B cells in pericardial adipose tissues were shown to improve, and their depletion worsen, myocardial injury and cardiac dysfunction in mice [162]. IL-10 was found to have potent anti-atherosclerotic effects, which was demonstrated by a 60% reduction in fatty lesions in diet-induced atherosclerosis in IL-10 knockout mice upon in vivo supplementation of IL-10 [163]. Such non-immunomodulatory effects of cytokines induced by vaccinations may also contribute to the observed PSEs (Table 1).

## 5. Vaccines: In Pursuit of Perfection

Vaccines are continually evolving as our understanding of the immune system and pathophysiology improves and new technologies are developed. Despite the vital role that vaccines play in human health, they can cause local reactions such as pain, redness, and moderate induration at the injection site due to the stimulation of inflammatory responses. Such reactogenic symptoms are common physical manifestations of an inflammatory reaction to a vaccine and can be a useful indicator of whether the vaccine is working. However, given that prophylactic vaccines are generally administered to healthy individuals, these mild and self-limiting adverse events are considered undesirable. When a vaccine is administered through a parenteral route, the conserved PAMP on the vaccine antigen is recognized by the first responders, macrophages and monocytes. These cells release inflammatory mediators, such as IL-1, IL-6, and TNFα, and recruit other immune cells to the site of injection [164]. These pro-inflammatory cytokines are well-known endogenous pyrogens and may cause moderate fever in vaccine recipients through a variety of mechanisms [165]. Stimulation of the innate immune response has also been observed when adjuvant alone is injected. As such, adjuvanted antigens in vaccines are expected to induce an amplified innate immune response compared to either component alone. Pyrogenic cytokines at the injection site are typically cleared within 72 h, and the recruited cells within 5–7 days, resulting in a transient reactogenicity to vaccines [166,167]. Professional antigen-processing cells, like macrophages and dendritic cells, process and present the injected vaccine antigens to T cells and B cells in the draining lymph nodes, triggering a cascade of antigen-specific acquired immune responses. As a result, the majority of vaccines, selected to meet an acceptable safety profile and to achieve an immune correlate of protection, generate a balanced immune response and offer protection from infections and the associated inflammatory reactions.

Occasionally, some vaccines have had unintended consequences in a small group of recipients for reasons that are often host-driven. For example, LAIV contains a cold-adapted live influenza virus that can replicate in the upper respiratory tract, like the pathogenic influenza virus. Children immunized with LAIV were observed to have an increased carriage density of *Streptococcus pneumoniae* and *Staphylococcus aureus* by 10 to 1000-fold, potentially by altering the nasal microbiota [168]. Although such an increase in bacterial colonization did not increase associated clinical disease, it may rise the transmission of off-target pathogens to vulnerable elderly and unvaccinated contacts [107]. A few pathogens are known to hijack the immune system in their favor, resulting in immune response-assisted disease exacerbation. The development of efficacious vaccines against such diseases has been met with many challenges [169,170]. Dengue virus (DENV) is one such example, whereby cross-reactive non-neutralizing antibodies induced after an initial DENV infection can facilitate the entry and replication of a different serotype of DENV in macrophages upon a second DENV infection. This phenomenon, known as antibody-dependent enhancement (ADE), can lead to severe dengue infection and death and is antibody concentration-dependent [171,172]. In individuals who had been exposed to DENV and were positive for DENV-specific antibodies (i.e., seropositive individuals), the dengue vaccine lowered severe virologically confirmed dengue and related hospitalization over 5 years by approximately 70% compared with control groups in the 2 to 16 years age group [173]. However, in seronegative individuals, the dengue vaccine mimicked a first DENV infection and exacerbated the disease when they encountered DENV at a later stage [173]. Following this discovery, the dengue vaccine is now only recommended for adolescents who have laboratory-confirmed evidence of previous dengue infection [174]. Similar issues delayed the development of vaccines against RSV disease. Meanwhile, the pneumococcal conjugate vaccine, which is highly effective against *Streptococcus pneumoniae*, resulted in a significant reduction in target strains but an increase in non-prevalent strains [175,176]. Such an effect demands the continued development of pneumococcal conjugate vaccines with higher valences [177]. Additionally, a vaccine developed against Lyme disease was found to induce a cross-reactive autoimmune response causing arthritis in genetically pre-disposed individuals [178]. Fears of vaccine side effects and the requirement for three vaccinations to evoke a protective response were among a number of factors that contributed to the withdrawal of the Lyme disease vaccine from the market [178]. Despite numerous successes, developing safe and effective vaccines against pathogens that trick the immune system has been challenging. Effective vaccines against these diseases will rely on upcoming advances in modern technologies and improved knowledge of the pathogenesis of these diseases.

Others have extensively discussed the modulation of innate immunity and the use of trained immunity to improve protection against certain pathogens, allergies, autoimmune diseases and cancers [10,11,12,179]. A few groups have proposed the use of trained immunity-based vaccines (TIvB) to protect hosts against a wide spectrum of pathogens by inducing anamnestic innate immunity through epigenetic changes in both mononuclear and polymorphonuclear myeloid cells [180,181,182,183]. These TIvB could be administered as a stand-alone vaccine or as an adjuvant to current vaccines designed to protect hosts against specific pathogens [184,185]. The application of innate immune responses to enhance protection against a broad spectrum of pathogens or specific pathogens warrants further investigation, given the current understanding of the immune system as a whole.

## 6. Conclusions

Many of the studies presented here demonstrate correlation and not causation of various effects, opening new areas for investigation. Nonetheless, the benefits of vaccinations that go above and beyond the call of duty are undeniable. The enormous benefits of vaccination on human health have been long established, from as far back as the early description of the inoculation of cowpox in the ancient Sanskrit writings, the *Sakteya Grantham* [186]. Vaccines have since saved millions of human lives from deadly diseases such as smallpox, poliomyelitis, diphtheria, tetanus, meningitis, mumps, pertussis, hepatitis B, influenza, chickenpox, COVID-19 and many more, some of which have been eradicated or are on the verge of being eradicated from the world. In this review, we describe several secondary benefits that are directly relevant to human health. The PSEs of vaccines have been demonstrated through various post hoc analyses or observational studies. Randomized clinical studies are needed to capture and refine our understanding of relevant PSEs of vaccines as secondary endpoints [187]. Aside from these benefits, the merit of vaccines on the economy and the emergence of new pathogens cannot be ignored. The reduction in vaccine-preventable diseases as a result of successful vaccination campaigns has diminished the costs of treating the diseases and of probable related absenteeism from work, resulting in significant economic benefits [188]. Vaccinations, particularly in developing countries, have also considerably reduced indiscriminate antibiotic usage, and hence antimicrobial resistance and the emergence of dangerous superbugs [188,189,190]. Vaccines have the potential to provide greater benefit than is currently recognized through the exploitation of the immune system. However, to ensure a global reach for all potential benefits of vaccination, including prevention of the potential emergence and spread of new variants, particularly during a pandemic, equitable vaccine distribution is required. Indeed, only timely vaccinations can help us realize the mantra, ‘the immune system trained keeps the pathogens restrained’.

## Figures and Tables

**Table 1:** The table shows the mechanisms of specific and para-specific protections by vaccination.

Vaccine-Induced Benefits	Potential Primary Mechanisms
1. Protect from specific disease (primary target)	Specific adaptive immunity
2. Cross-protect against closely related strains, subtypes, and subgroups	Cross-reactivity of adaptive immunity effectors with conserved epitopes
3. Prevent potential cytokine storms and related complications	Elimination or reduction of target pathogen load from the system Induction of Goldilocks immunity with checks and balances on each type of response, resulting in a ‘quiet alert’ state of the immune system
4. Abate exacerbation of pre-existing conditions	Elimination or lessening of the burden from the primary disease and the associated secondary complications such as tissue damage, cytokine storm, inflammation etc. Dampening of the inflammatory or allergic mediators with counteracting mediators
5. Reduce susceptibility to unrelated pathogens, autoimmune diseases, and pre-existing conditions	Non-specific protection through trained immunity Prevention, through a balanced response, of pre-eminence of one arm over the other and the associated complications. Non-immunomodulatory effects of cytokines on other tissues improving their conditions
6. Reduced secondary bacterial complications	Prevention of primary viral infections eliminates viral-mediated immune suppression such as the killing of alveolar macrophages and impairment of natural killer cells
